# Updated Estimates of Agreement Between Dispensed Medications and Forensic Toxicology in Sweden

**DOI:** 10.1002/pds.70483

**Published:** 2026-09-24

**Authors:** Heidi Taipale, Jari Tiihonen, Antti Tanskanen, Jonas Forsman

**Affiliations:** ^1^ School of Pharmacy, University of Eastern Finland, Kuopio, Finland; Department of Clinical Neuroscience Karolinska Institutet Stockholm Sweden; ^2^ Department of Clinical Neuroscience, Karolinska Institutet, Stockholm, Sweden; Department of Forensic Psychiatry Niuvanniemi Hospital Kuopio Finland; ^3^ Department of Clinical Neuroscience, Karolinska Institutet, Stockholm, Sweden; Department of Forensic Psychiatry National Board of Forensic Medicine Huddinge Sweden

**Keywords:** cardiovascular agents, drug utilization, forensic toxicology, pharmacoepidemiology, prescription registry, psychotropic drugs, validation

## Abstract

**Purpose:**

To present updated estimates from a nationwide Swedish study comparing dispensed medications with forensic toxicological findings (2006–2013), following re‐evaluation of the toxicology dataset to address periods with non‐uniform analytical detection and inconsistent testing.

**Methods:**

Observations from time periods with non‐uniform detection or inconsistent testing were excluded. Analyses were repeated using the PRE2DUP method to model drug exposure. Agreement between PRE2DUP‐modelled exposure and post‐mortem toxicology was assessed using sensitivity, specificity, predicted adherence, and Cohen's *κ*.

**Results:**

After exclusion of inconsistent testing periods, the number of individuals for affected substances was reduced (10 060 vs. 18 627). Proportional changes in sensitivity and specificity were negligible. In contrast, predicted adherence and Cohen's *κ* improved across several substances. Moderate to substantial proportional increases were observed for selected medications but translated into only small group‐level effects (approximately 2%–7%). The largest improvements were observed for cardiovascular drugs, beta‐blockers, for which Cohen's *κ* and predicted adherence increased substantially (approximately 40%–55%) at the class level; notably, only for these substances did Cohen's *κ* shift interpretation (from fair to moderate agreement). Overall, proportional improvements across all substances were moderate (approximately 10%–15%). Despite these changes, overall agreement patterns remained comparable to the original results.

**Conclusions:**

After exclusion of periods with non‐uniform or incomplete toxicological testing, agreement between PRE2DUP‐modelled exposure and forensic toxicology improved overall. Moderate increases observed for a few individual medications translated into only small effects at the group level, except for cardiovascular drugs, for which improvements were substantial. These updates do not materially alter the original interpretation but strengthen the validity of register‐based drug exposure modelling.

## Key Points

- Updated nationwide analyses showed that excluding periods with inconsistent toxicological testing modestly improved agreement between PRE2DUP‐modelled drug exposure and forensic‐toxicological findings without materially changing the original conclusions.
- The largest improvements were observed for cardiovascular drugs, where agreement estimates increased substantially after restricting analyses to periods with uniform testing.
- Measures of classification changed minimally, supporting the robustness of PRE2DUP exposure modelling.
- Findings suggest that some previously observed discrepancies reflected methodological variation in toxicological analyses rather than true disagreement between prescription‐based exposure estimates and biological detection.
- The results strengthen confidence in PRE2DUP for register‐based pharmacoepidemiological exposure modelling.

## Plain Language Summary

This study re‐examined data from a nationwide Swedish study comparing prescription records with substances detected in forensic toxicology after death. Researchers used a method called PRE2DUP, which mathematically estimates whether a person was likely taking a medication based on prescription records. After discovering that some medications had not been tested consistently throughout the original study period, the researchers repeated the analyses using only time periods with identical toxicological testing. Overall, the updated analyses showed slightly better agreement between prescription‐based estimates and toxicology findings, while the main conclusions of the original study remained unchanged. The largest improvements were seen for cardiovascular drugs, where earlier inconsistencies appeared partly related to differences in laboratory testing methods rather than inaccuracies in prescription data. Measures showing how well the method identified both medication users and non‐users changed very little, supporting the overall reliability of the approach. These findings increase confidence that prescription records and PRE2DUP‐based exposure modelling can be used to estimate which medications people were likely taking, while also showing that differences in laboratory testing methods can affect how accurately medication use is measured.

## 1 Introduction

Register‐based pharmacoepidemiological research relies on accurate modelling of medication exposure from dispensing data. The PRE2DUP method reconstructs continuous drug use periods based on dispensing patterns and dosage information [1]. In the original Swedish study (*Comparison of dispensed medications and forensic‐toxicological findings to assess pharmacotherapy in the Swedish population 2006 to 2013*), PRE2DUP‐modelled medication exposure was compared with forensic‐toxicological findings from systematic post‐mortem examinations in Sweden to evaluate concordance between register‐based exposure modelling and biological detection [2]. Subsequent review of the toxicological dataset identified periods where analytical detection limits varied across laboratory methods and over time, and where certain substances were not consistently tested throughout the study period. To ensure methodological consistency and to explore potential improvements in PRE2DUP's predictive performance, observations of substances originating from periods without consistent testing were excluded and analyses repeated. The present report provides updated results and describes proportional changes of measures and improvements of agreement relative to the original publication and suggestions for practical use and further research [2].

## 2 Methods

The study population and register linkages were identical to those described in the original nationwide analysis [2]. In brief, data on all forensically investigated deaths in Sweden were obtained from the Swedish National Board of Forensic Medicine (NBFM) toxicology database and linked at the individual level to nationwide registers held by the National Board of Health and Welfare, including the Swedish National Prescription Registry and the National Patient Registry [3, 4, 5]. The prescription registry contains complete information on all dispensed medications in Sweden since July 2005, while the patient registry provides data on inpatient care since 1987. The study sample comprised individuals with complete information on demographic characteristics, cause of death, date of death, toxicological findings, and dispensed prescriptions. Consistent with the original study, individuals with self‐inflicted deaths and those with recent inpatient or ambulance care or death were excluded to minimize misclassification of drug exposure due to non‐recorded in‐hospital medication use. Dispensed medication data were used to model continuous drug exposure periods using the PRE2DUP method, a validated algorithm that reconstructs drug use based on individual dispensing patterns, accounting for stockpiling, dosage, and package information [6]. The method applies sliding averages of defined daily doses and evaluates temporal purchasing behavior to estimate likely periods of active drug use. As the analysis focused on exposure at the time of death, the most recent dispensing periods were of primary relevance. Toxicology data obtained from NBFM's toxicology database was based on results from routinely performed analyses using accredited methods, including liquid chromatography–time‐of‐flight screening followed by confirmatory analyses with mass spectrometry [7]. The toxicological panel covers a broad range of substances and has since September 2011 been applied systematically across cases, providing an objective biological indicator of recent drug exposure [8].

**FIGURE 1:**
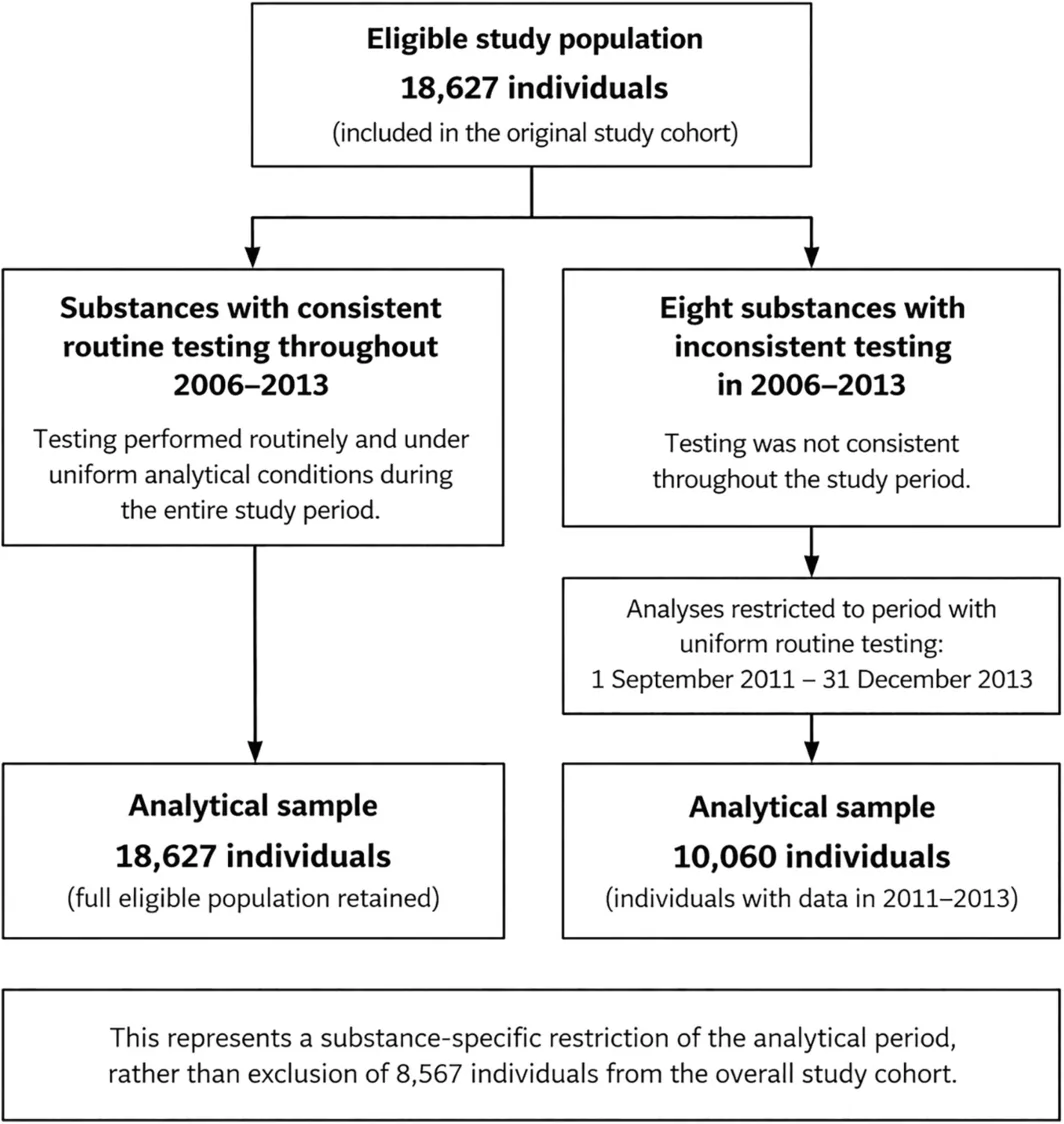
Derivation of substance‐specific analytical samples according to consistency of routine toxicological testing during the study period.

In the present updated analysis, observations originating from time periods with non‐uniform analytical detection limits or inconsistent testing of specific substances were excluded to ensure comparability across the study period (2006–2013). For eight substances, analyses were therefore restricted to the period with consistent routine testing (1 September 2011–31 December 2013), comprising 10 060 individuals, whereas the full eligible population of 18 627 individuals was retained for substances with consistent testing throughout 2006–2013. The derivation of these substance‐specific analytical samples is shown in Figure 1, with further details provided in Table S3. Analyses were then repeated using the same modelling framework as in the original study.

Agreement between PRE2DUP‐modelled drug exposure and toxicological findings was assessed at the individual level. Toxicological detection served as the reference standard. Measures of agreement included sensitivity, specificity, predicted adherence (the fraction of dispensed prescriptions matching positive toxicology), and Cohen's *κ* with 95% confidence intervals. At the substance level, each individual contributed at most one observation per substance. Class‐level and overall agreement measures were calculated by pooling substance‐specific observations; consequently, the same individual could contribute observations for more than one substance to these aggregate estimates. Cohen's *κ* was interpreted using conventional thresholds (poor to very good agreement). All analyses were conducted consistently with the original analytical approach.

## 3 Results

After applying revised inclusion criteria excluding all observations from periods with non‐uniform analytical detection limits or inconsistent testing, the number of individuals included in analyses for affected substances was reduced to 10 060, compared with 18 627 for substances with consistent testing throughout the study period (Table 1). Across all investigated substances, proportional changes (%) in sensitivity and specificity were negligible, indicating that classification performance remained stable following dataset refinement. In contrast, changes in predicted adherence and Cohen's *κ* were more pronounced and therefore constitute the primary focus of the updated analyses.

**TABLE 1:** Agreement between registry‐based drug use modelled with PRE2DUP and forensic‐toxicological findings (including new estimates in **bold** ^b^).

Drug substance (ATC)	Both positive	PRE2DUP negative, toxicology positive	Both negative	PRE2DUP positive, toxicology negative	Sensitivity	Sensitivity (original paper)	Prop. change sensitivity	Specificity	Specificity (original paper)
	True‐positives	False‐negatives	True‐negatives	False‐positive					
Antidepressants									
Citalopram (N06AB04)^a^	639	170	17 662	156	0.790	0.790	0.0%	0.991	0.991
Clomipramine (N06AA04)^a^	63	15	18 537	12	0.808	0.808	0.0%	0.999	0.999
Duloxetine (N06AX21)^b^	**42**	**11**	**9971**	**36**	**0.792**	0.806	**−1.7%**	**0.996**	0.997
Fluoxetine (N06AB03)^a^	61	28	18 504	34	0.685	0.685	0.0%	0.998	0.998
Mirtazapine (N06AX11)^a^	362	98	17 953	214	0.787	0.787	0.0%	0.988	0.988
Paroxetine (N06AB05)^b^	**44**	**8**	**9973**	**35**	**0.846**	0.829	**2.1%**	**0.997**	0.960
Sertraline (N06AB06)^a^	243	64	18 241	79	0.792	0.792	0.0%	0.996	0.996
Venlafaxine (N06AX16)^a^	203	52	18 310	62	0.796	0.796	0.0%	0.997	0.997
Total antidepressants	**1657**	**446**	**129 151**	**628**	0.788	0.788	**0.0%**	0.995	0.995
Antiepileptics									
Carbamazepine (N03AF01)^a^	279	148	18 137	63	0.653	0.653	0.0%	0.997	0.997
Gabapentin (N03AX12)^a^	49	19	18 476	83	0.721	0.721	0.0%	0.996	0.996
Lamotrigine (N03AX09)^a^	118	19	18 459	31	0.861	0.861	0.0%	0.998	0.998
Pregabalin (N03AX16)^b^	**142**	**109**	**9658**	**151**	**0.566**	0.582	**2.7%**	**0.985**	0.998
Topiramate (N03AX11)^a^	26	4	18 590	7	0.867	0.867	0.0%	1000	1000
Total antiepileptics	**614**	**299**	**83 320**	**335**	**0.673**	0.676	**0.4%**	0.996	0.996
Antipsychotics									
Clozapine (N05AH02)^a^	49	2	18 576	0	0.961	0.961	0.0%	1000	1000
Haloperidol (N05AD01)^b^	**27**	**12**	**9999**	**22**	**0.692**	0.739	**−6.4%**	**0.998**	0.997
Olanzapine (N05AH03)^a^	153	30	18 364	80	0.836	0.836	0.0%	0.996	0.996
Quetiapine (N05AH04)^a^	54	13	18 492	68	0.806	0.806	0.0%	0.996	0.996
Total antipsychotics	**283**	**57**	**65 431**	**170**	**0.832**	0.836	**−0.5%**	0.997	0.997
Cardiovascular drugs									
Atenolol (C07AB03)^b^	**150**	**29**	**9674**	**207**	**0.838**	0.833	**0.6%**	**0.979**	0.969
Bisoprolol (C07AB07)^b^	**92**	**18**	**9715**	**235**	**0.836**	0.836	**0.0%**	**0.976**	0.978
Metoprolol (C07AB02)^b^	**324**	**67**	**8996**	**691**	**0.829**	0.824	**0.6%**	**0.929**	0.925
Propranolol (C07AA05)^b^	**45**	**13**	**9881**	**121**	**0.776**	0.747	**3.9%**	**0.988**	0.988
Verapamil (C08DA01)^a^	33	9	18 559	26	0.786	0.786	0.0%	0.999	0.999
Total cardio‐vascular drugs	**644**	**136**	**56 826**	**1280**	**0.826**	0.818	**1.0%**	**0.978**	0.972
Sedatives (as needed)									
Alimemazine (R06AD01)^a^	309	243	17 890	185	0.560	0.560	0.0%	0.990	0.990
Hydroxyzine (N05BB01)^a^	183	90	18 091	263	0.670	0.670	0.0%	0.986	0.986
Promethazine (R06AD02)^a^	113	71	18 323	120	0.614	0.614	0.0%	0.993	0.993
Propiomazine (N05CM06)^a^	561	196	17 353	517	0.741	0.741	0.0%	0.971	0.971
Zolpidem (N05CF02)^a^	194	67	17 859	507	0.743	0.743	0.0%	0.972	0.972
Total sedatives	1360	667	89 516	1592	0.671	0.671	0.0%	0.983	0.983
**Total all substances**	**4558**	**1605**	**424 260**	**4004**	**0.740**	**0.741**	**0.1%**	**0.991**	0.989

Drug substance (ATC)	Prop. change specificity	Predicted adherence	Predicted adherence (original paper)	Prop. change predicted adherence	Recreational use	Cohen's kappa (95% CI)	Cohen's kappa (95% CI) (original paper)	Prop. Change Cohen's kappa
Antidepressants								
Citalopram (N06AB04)^a^	0.0%	80.4%	80.40%	0.0%	1.0%	0.79 (0.77–0.81)	0.79 (0.77**–**0.81)	0.0%
Clomipramine (N06AA04)^a^	0.0%	84.0%	84.0%	0.0%	0.1%	0.82 (0.76–0.89)	0.82 (0.76**–**0.89)	0.0%
Duloxetine (N06AX21)^b^	**−0.1%**	**53.8%**	**46.30%**	**16.2%**	**0.1%**	**0.64 (0.54–0.74)**	**0.59 (0.49–0.68)**	**8.5%**
Fluoxetine (N06AB03)^a^	0.0%	64.2%	64.2%	0.0%	0.2%	0.66 (0.58–0.75)	0.66 (0.58**–**0.75)	0.0%
Mirtazapine (N06AX11)^a^	0.0%	62.8%	62.8%	0.0%	0.5%	0.69 (0.66–0.72)	0.69 (0.66**–**0.72)	0.0%
Paroxetine (N06AB05)^b^	**3.9%**	**55.7%**	**46.00%**	**21.1%**	**0.1%**	**0.67 (0.57–0.77)**	**0.59 (0.50‐0.68)**	**13.6%**
Sertraline (N06AB06)^a^	0.0%	75.5%	75.5%	0.0%	0.3%	0.77 (0.73–0.81)	0.77 (0.73**–**0.81)	0.0%
Venlafaxine (N06AX16)^a^	0.0%	76.6%	76.6%	0.0%	0.3%	0.78 (0.74–0.82)	0.78 (0.74**–**0.82)	0.0%
Total antidepressants	0.0%	**72.5%**	**71.00%**	**2.1%**	0.3%	**0.75 (0.74–0.77)**	**0.74 (0.73–0.76)**	**1.4%**
Antiepileptics								
Carbamazepine (N03AF01)^a^	0.0%	81.6%	81.6%	0.0%	0.8%	0.72 (0.68–0.76)	0.72 (0.68**–**0.76)	0.0%
Gabapentin (N03AX12)^a^	0.0%	37.1%	37.1%	0.0%	0.1%	0.49 (0.39–0.59)	0.49 (0.39**–**0.59)	0.0%
Lamotrigine (N03AX09)^a^	0.0%	79.2%	79.2%	0.0%	0.1%	0.82 (0.78–0.87)	0.82 (0.78**–**0.87)	0.0%
Pregabalin (N03AX16)^b^	**1.3%**	**48.5%**	**40.30%**	**20.3%**	**1.1%**	**0.51 (0.45–0.57)**	**0.47 (0.41–0.52)**	**8.5%**
Topiramate (N03AX11)^a^	0.0%	78.8%	78.8%	0.0%	0.0%	0.83 (0.72–0.93)	0.83 (0.72**–**0.93)	0.0%
Total antiepileptics	**0.0%**	**64.7%**	**60.30%**	**7.3%**	**0.4%**	**0.66 (0.63–0.68)**	**0.63 (0.61–0.66)**	**4.8%**
Antipsychotics								
Clozapine (N05AH02)^a^	0.0%	100%	100%	0.0%	0.0%	0.98 (0.95–1.01)	0.98 (0.95–1.01)	0.0%
Haloperidol (N05AD01)^b^	**0.1%**	**55.1%**	**37.00%**	**48.9%**	**0.1%**	**0.61 (0.48–0.74)**	**0.49 (0.37**–**0.61)**	**24.5%**
Olanzapine (N05AH03)^a^	0.0%	65.7%	65.7%	0.0%	0.2%	0.73 (0.68–0.78)	0.73 (0.68–0.78)	0.0%
Quetiapine (N05AH04)^a^	0.0%	44.3%	44.3%	0.0%	0.1%	0.57 (0.48–0.66)	0.57 (0.48–0.66)	0.0%
Total antipsychotics	**0.0%**	**62.5%**	**58.50%**	**6.8%**	0.1%	**0.71 (0.68–0.75)**	**0.69 (0.65**–**0.72)**	**2.9%**
Cardiovascular drugs								
Atenolol (C07AB03)^b^	**1.0%**	**42.0%**	**21.30%**	**97.2%**	**0.3%**	**0.55 (0.49–0.61)**	**0.33 (0.28–0.38)**	**66.7%**
Bisoprolol (C07AB07)^b^	**−0.2%**	**28.1%**	**18.40%**	**52.7%**	**0.2%**	**0.41 (0.34–0.48)**	**0.29 (0.23–0.36)**	**41.4%**
Metoprolol (C07AB02)^b^	**0.4%**	**31.9%**	**21.30%**	**49.8%**	**0.7%**	**0.43 (0.39–0.47)**	**0.31 (0.28–0.35)**	**38.7%**
Propranolol (C07AA05)^b^	**0.0%**	**27.1%**	**22.00%**	**23.2%**	**0.1%**	**0.40 (0.30–0.50)**	**0.34 (0.25–0.42)**	**17.6%**
Verapamil (C08DA01)^a^	0.0%	55.9%	55.9%	0.0%	0.0%	0.65 (0.54–0.77)	0.65 (0.54–0.77)	0.0%
Total cardio‐vascular drugs	**0.6%**	**33.5%**	**21.50%**	**55.8%**	**0.2%**	**0.47 (0.44–0.49)**	**0.33 (0.31‐0.36)**	**42.4%**
Sedatives (as needed)								
Alimemazine (R06AD01)^a^	0.0%	62.6%	62.6%	0.0%	1.3%	0.58 (0.54–0.62)	0.58 (0.54–0.62)	0.0%
Hydroxyzine (N05BB01)^a^	0.0%	41.0%	41.0%	0.0%	0.5%	0.50 (0.45–0.55)	0.50 (0.45–0.55)	0.0%
Promethazine (R06AD02)^a^	0.0%	48.5%	48.5%	0.0%	0.4%	0.54 (0.47–0.60)	0.54 (0.47–0.60)	0.0%
Propiomazine (N05CM06)^a^	0.0%	52.0%	52.0%	0.0%	1.1%	0.59 (0.56–0.62)	0.59 (0.56–0.62)	0.0%
Zolpidem (N05CF02)^a^	0.0%	27.7%	27.7%	0.0%	0.4%	0.39 (0.34–0.44)	0.39 (0.34–0.44)	0.0%
Total sedatives	0.0%	46.1%	46.1%	0.0%	0.7%	0.53 (0.52–0.55)	0.53 (0.52–0.55)	0.0%
**Total all substances**	**0.2%**	**53.2%**	**46.0%**	**15.7%**	**0.4%**	**0.61 (0.60–0.62)**	**0.56 (0.55–0.57)**	**8.9%**

*Note:* Agreement between registry‐based drug use modelled with PRE2DUP and forensic‐toxicological findings (including new estimates in bold).

^a^ Routine method “G1/G2” 2006‐01‐01–2011‐08‐31; “T1”, 2011‐09‐01–2013‐12‐31.

^b^ Method T1, Substances were included only during this period due to the absence of routine testing at other times.

Among the antidepressants, duloxetine and paroxetine showed moderate increases in predicted adherence and Cohen's *κ* (16%–21% and 9%–14%, respectively) following exclusion of inconsistent testing periods. A similar pattern was observed for the antiepileptic pregabalin (9%–20%), whereas the antipsychotic haloperidol showed a moderate to substantial improvement for Cohen's *κ* (25%) and predicted adherence (49%). The most substantial changes, however, were observed among cardiovascular drugs—beta‐blockers—including atenolol, bisoprolol, metoprolol, and propranolol. For these substances, predicted adherence and Cohen's *κ* increased markedly, with overall proportional improvements exceeding those observed in other drug classes (23%–97% and 18%–67%, respectively). Notably, only for cardiovascular drugs did the magnitude of change in Cohen's *κ* result in a shift in the level of interpretation, from fair to moderate agreement; no such change in interpretative category was observed for other substances. At group level, moderate increases in predicted adherence and Cohen's *κ* observed for several individual substances resulted in only small overall changes (approximately 2%–7%), with the exception of cardiovascular drugs, for which improvements were substantial (predicted adherence 56% and Cohen's *κ* 42%). The 95% confidence intervals for Cohen's *κ* remained of similar width for all substances. Despite these updates, the overall pattern of agreement between PRE2DUP‐modelled exposure and forensic toxicological findings remained consistent with the original results.

**FIGURE 2:**
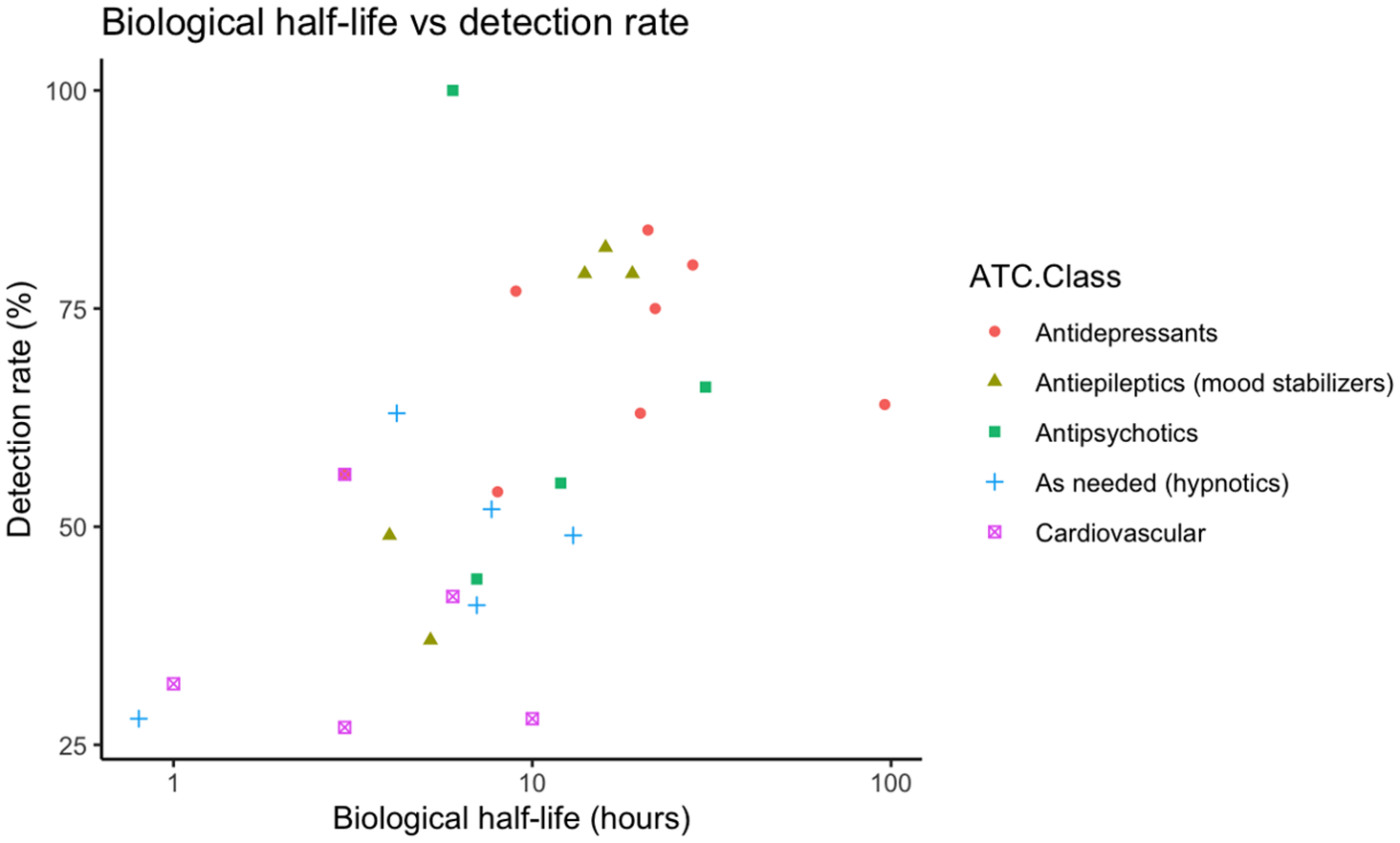
Biological half‐life versus detection rate. Detection rate is plotted against the known biological half‐life of each substance (see Table S1 for pharmacokinetic properties and detection thresholds). A generally positive linear relationship is observed for most substances, whereas cardiovascular drugs now show no clear relationship.

The relationship between biological half‐life and detection rate of PRE2DUP (predicted adherence) provides additional insight into the mechanisms underlying agreement (Figure 2). As in the original analysis, a generally positive association was observed for most substances, indicating that drugs with shorter half‐lives are less likely to be detected upon toxicology. Notably, this relationship remained largely unchanged after exclusion of inconsistent testing periods, reinforcing the role of pharmacokinetic factors in determining detectability. In contrast, cardiovascular drugs now show no clear association, with consistently low detection rates across the half‐life spectrum. This pattern strengthens the interpretation that the discrepancy between prescription‐based estimates and toxicology‐based detection in this drug class is not explained by half‐life alone, but likely reflects factors such as adherence patterns, intermittent dosing, or clinical use characteristics.

## 4 Discussion

In this updated nationwide analysis of agreement between dispensed medications, operationalized through PRE2DUP‐modelled drug exposure, and forensic‐toxicological findings in Sweden, exclusion of observations from periods with non‐uniform analytical detection limits or inconsistent testing led to modest but consistent improvements in agreement estimates. Importantly, these refinements did not materially alter the overall pattern of results reported in the original study but instead strengthen the evidence supporting the validity of PRE2DUP for modelling continuous drug use in register‐based research [2]. A similar pattern of modest numerical changes without impact on overall interpretation has been observed in a related re‐evaluation of toxicological data in a nationwide case–control study of psychotropic medication adherence in completed suicide, further supporting the robustness of findings derived from PRE2DUP‐modelled exposure despite earlier analytical inconsistencies [9].

Consistent with the original findings of the present nationwide comparison, agreement between modelled exposure and toxicology remained moderate overall, with clear variation across drug classes. In the original study, predicted adherence was highest for antidepressants and lowest for cardiovascular drugs, with corresponding differences in Cohen's *κ*. The updated analyses confirm this pattern but demonstrate that part of the previously observed discrepancy—particularly for certain substances—can be attributed to inconsistencies in toxicological detection over time. After restricting analyses to periods with uniform testing, predicted adherence and agreement improved across several substances, most notably for cardiovascular drugs, where substantial increases were observed at both the substance and class level, although levels remained lower than those observed for other drug classes.

These findings suggest that earlier estimates partly reflected methodological artefacts rather than true disagreement between prescription‐based modelling and biological detection. At the same time, the persistence of relatively lower agreement for cardiovascular drugs, even after correction, indicates that methodological factors alone do not fully explain the discrepancy. Notably, this pattern was consistent in analyses restricted to cardiovascular deaths (Table S2), suggesting that it is not driven by underlying case‐mix but likely reflects factors such as adherence behavior, intermittent use, or dosing patterns below toxicological detection thresholds.

An important limitation concerns the use of postmortem toxicology as a single‐time‐point measure of drug exposure. Detectability depends not only on ongoing medication use but also on the interval between the last dose and death, drug‐specific elimination half‐life and detection thresholds, and individual pharmacokinetic characteristics. Furthermore, postmortem redistribution may alter measured blood concentrations for some substances. Consequently, a negative toxicological finding does not necessarily indicate non‐adherence, particularly for drugs with short elimination half‐lives or when the interval since the last dose is prolonged. A further limitation concerns the class‐level and overall estimates, for which observations were pooled across substances and the same individual could therefore contribute more than one observation. Confidence intervals for pooled Cohen's *κ* estimates were calculated without adjustment for within‐person clustering and may consequently be somewhat narrower than cluster‐adjusted intervals. This does not apply to the substance‐specific estimates, for which each individual contributed at most one observation per substance.

Future work should focus on replication using more recent data with harmonized toxicological methods, enabling clearer separation of methodological artefacts from true adherence patterns. In parallel, refinement of the PRE2DUP algorithm—particularly through incorporation of pharmacokinetic properties, intermittent use patterns, and individual‐level variability—may improve performance for drug classes with persistently lower agreement. Such developments would strengthen the validity and interpretability of register‐based exposure modelling in pharmacoepidemiological research.

## Author Contributions

H.T., A.T., J.T., and J.F. drafted and critically revised the paper. H.T., A.T., and J.F. formulated the research question and designed the study. J.F. ran the project management, including ethical approval, and data collection. H.T., A.T., and J.F. conducted the analyses.

## Funding

The authors have nothing to report.

## Ethics Statement

In this registry‐based postmortem study, all personal information was anonymized, rendering unnecessary the need for formal ethical approval. The study was nonetheless reviewed and approved by the Regional Ethical Review Board in Stockholm (2013/1411‐31/5).

## Conflicts of Interest

H.T. reports personal fees from Gedeon Richter, Janssen, Lundbeck, Otsuka and Teva. J.T. has participated in research projects funded by grants from Janssen, EU/Forte, Finnish State Research Funding, and Jane and Aatos Erkko Foundation to his employing institutions; has been a consultant to and/or member of Advisory Board to and/or has received honoraria from and/or given expert testimony for and/or received support for attending meetings from: Healthcare Global Village, Janssen, Lundbeck, Orion Pharma, Otsuka, and Teva; all outside the submitted work. The other authors declare no conflicts of interest.

All studied medicinal products in this paper were generic substances without exclusive ownership by any company.

## Supporting information


**Table S1:** Elimination half‐lives, detection thresholds, therapeutic ranges and postmortem redistribution factors for the studied drugs.
**Table S2:** Agreement between registry‐based data on drug‐use modeled with PRE2DUP and toxicology findings among cardiovascular deaths (ICD10: I00‐I99).

**Table S3:** Supporting Information.

## Data Availability

The data supporting the findings of this study are not publicly available because they contain sensitive personal and clinical information. Data sharing is restricted to protect participant confidentiality and to comply with ethical approvals and the General Data Protection Regulation (GDPR).
