# Supplementary material for: Updated Estimates of Agreement Between Dispensed Medications and Forensic Toxicology in Sweden
Source: Pharmacoepidemiol Drug Saf. 2026 Sep 24;35(10):e70483. doi: 10.1002/pds.70483 (PMC13602084; doi:10.1002/pds.70483)
Supplement: Supplementary file 1 [file PDS-35-e70483-s001.docx]

| **Supplementary Table 3.** | | | | |  |  |
| --- | --- | --- | --- | --- | --- | --- |
| **Substance** | **ATC** | **ATC class** | **Period included in updated analysis** | **Reason for restriction** |  |  |
|  |  |  |  |  |  |  |
| duloxetine | N06AX21 | Antidepressants | 2011-09-01–2013-12-31 | Not routinely tested during the earlier period |  |  |
| paroxetine | N06AB05 | Antidepressants | As above | As above |  |  |
| pregabalin | N03AX16 | Antiepileptics | As above | As above |  |  |
| haloperidol | N05AD01 | Antipsychotics | As above | As above |  |  |
| atenolol | C07AB03 | Beta-blocking agents | As above | As above |  |  |
| bisoprolol | C07AB07 | Beta-blocking agents | As above | As above |  |  |
| metoprolol | C07AB02 | Beta-blocking agents | As above | As above |  |  |
| propranolol | C07AA05 | Beta-blocking agents | As above | As above |  |  |
|  |  |  |  |  |  |  |
