# Supplementary material for: Updated Estimates of Agreement Between Dispensed Medications and Forensic Toxicology in Sweden
Source: Pharmacoepidemiol Drug Saf. 2026 Sep 24;35(10):e70483. doi: 10.1002/pds.70483 (PMC13602084; doi:10.1002/pds.70483)
Supplement: Supplementary file 2 [file PDS-35-e70483-s002.docx]

| **Updated Supplementary Table 1. Elimination half-lives, detection thresholds, therapeutic ranges and postmortem redistribution factors for the studied drugs.** | | | | | | |
| --- | --- | --- | --- | --- | --- | --- |
| **Substance** | **ATC^†^** | **ATC class^†^** | **Minimum elimination half-life (h) ^‡^** | **Detection threshold ^§^ (mg/L) *** | **Therapeutic range** ^\|\|^  **(mg/L)** | **Post-mortal redistribution factor** ^¶^ ^#^ |
|  |  |  |  |  |  |  |
| citalopram | N06AB04 | Antidepressant | 28.0 | 0.05 | 0.05-0.11 |  |
| clomipramine | N06AA04 | Antidepressant | 21.0 | 0.05 | 0.02-0.40 | 1.9 ^¶^; 58 ^#^ |
| duloxetine ** | N06AX21 | Antidepressant | 8.0 | 0.03 | 0.03-0.12 |  |
| fluoxetine | N06AB03 | Antidepressant | 96.0 | 0.02-0.10 | 0.12-0.50 |  |
| mirtazapine | N06AX11 | Antidepressant | 20.0 | 0.02-0.05 | 0.03-0.50 |  |
| paroxetine ** | N06AB05 | Antidepressant | 3.0 | 0.01-0.10 | 0.01-0.05 |  |
| Sertraline | N06AB06 | Antidepressant | 22.0 | 0.05 | 0.05-0.25 | 97.0 ^#^ |
| venlafaxine | N06AX16 | Antidepressant | 9.0 | 0.05 | 0.10-0.40 |  |
| carbamazepine | N03AF01 | Antiepileptic (mood stabilizers) | 16.0 | 0.50 | 2.00-8.00 |  |
| gabapentin | N03AX12 | Antiepileptic (mood stabilizers) | 5.2 | 0.50 | 0.50-6.00 |  |
| lamotrigine | N03AX09 | Antiepileptic (mood stabilizers) | 14.0 | 0.50 | 1.00-14.00 |  |
| pregabalin ** | N03AX16 | Antiepileptic (mood stabilizers) | 4.0 | 0.50 | 2.00-5.00 |  |
| opiramate | N03AX11 | Antiepileptic (mood stabilizers) | 19.0 | 2.00 | 2.00-10.00 |  |
| clozapine | N05AH02 | Antipsychotic | 6.0 | 0.05 | 0.10-0.60 |  |
| haloperidol ** | N05AD01 | Antipsychotic | 12.0 | 0.01 | 0.005-0.017 | 3.6 ^¶^ |
| olanzapine | N05AH03 | Antipsychotic | 30.4 | 0.02-0.05 | 0.02-0.08 |  |
| quetiapine | N05AH04 | Antipsychotic | 7.0 | 0.02-0.05 | 0.10-0.50 |  |
| atenolol ** | C07AB03 | Cardiovascular system | 6.0 | 0.02 | 0.15-0.50 |  |
| bisoprolol ** | C07AB07 | Cardiovascular system | 10.0 | 0.02 | 0.01-0.10 |  |
| metoprolol ** | C07AB02 | Cardiovascular system | 1.0 | 0.02 | 0.03-0.50 |  |
| popranolol ** | C07AA05 | Cardiovascular system | 3.0 | 0.02 | 0.02-0.30 |  |
| verapamil | C08DA01 | Cardiovascular system | 3.0 | 0.05-0.10 | 0.10-0.25 |  |
| alimemazine | R06AD01 | Sedative (As needed) | 4.2 | 0.05 | 0.05-0.40 |  |
| hydroxizine | N05BB01 | Sedative (As needed) | 7.0 | 0.03 | 0.05-0.10 |  |
| promethazine | R06AD02 | Sedative (As needed) | 13.0 | 0.05 | 0.05-0.20 |  |
| propiomazine | N05CM06 | Sedative (As needed) | 7.7 | 0.03 | 0.3-unknown |  |
| zolpidem | N05CF02 | Sedative (As needed) | 0.8 | 0.02-0.05 | 0.08-0.15 |  |
|  |  |  |  |  |  |  |

* Routine method “G1/G2” 2006-01-01—2011-08-31; “T1”, 2011-09-01--2013-12-31
** Method T1, Substances were included only during this period due to the absence of routine testing at other times.
† ATC: Anatomical Therapeutic Chemical

‡ Half-lives as officially reported by drug manufacturers to Läkemedelsindustriföreningen (<http://www.fass.se> 2017-04-05).
§ Detection thresholds (micrograms/ml = mg/L) with the use of LC-QTOF in instrument by the National Board of Forensic Medicine’s toxicological unit in Linköping, Sweden: (Valideringsbeskrivning för metod T01, B-läkemedel och droger. Ledningssystem För Rättsgenetik Och Rättskemi Dokument VT001 2016).
|| Therapeutic ranges Schulz, M., Iwersen-Bergmann, S., Andresen, H., & Schmoldt, A. (2012). Therapeutic and toxic blood concentrations of nearly 1,000 drugs and other xenobiotics. *Critical Care*, *16*(4), R136. <http://doi.org/10.1186/cc11441>
¶ Jerrold B. Leikin M.D., & William A. Watson Pharm.D. (2003) Post-mortem Toxicology: What The Dead Can And Cannot Tell Us, Journal of Toxicology: Clinical Toxicology, 41:1, 47-56, DOI: 10.1081/CLT-120018270
\# McIntyre. Liver and peripheral blood concentration ratio (L/P) as a marker of postmortem drug redistribution: a literature review, Forensic Sci Med Pathol (2014) 10:91–96, DOI 10.1007/s12024-013-9503-x

|  | **Updated Supplementary Table 2. Agreement between registry-based data on drug-use modeled with PRE2DUP and toxicology findings among cardiovascular deaths (ICD10: I00-I99)** | | | | | | | | | | | | | | | | | | |  |
| --- | --- | --- | --- | --- | --- | --- | --- | --- | --- | --- | --- | --- | --- | --- | --- | --- | --- | --- | --- | --- |
| **Drug substance  (ATC code)** **^†^** | | | **Both positive** | | **PRE2DUP negative, toxicology positive** | | **Both negative** | | **PRE2DUP positive, toxicology negative** | | **Sensitivity** | | **Specificity** | | **Predicted adherence** | | **Recreational use** | | **Cohen’s kappa (95% CI)** | |
|  |  |  |  |  |  |  |  |  |  |  |  |  |  |  |  |  |  |  |  |  |
|  |  |  |  |  |  |  |  |  |  |  |  |  |  |  |  |  |  |  |  |  |
|  |  |  | **True- positives** | | **False-negatives** | | **True-negatives** | | **False-positives** | |  |  |  |  |  |  |  |  |  |  |
| **Antidepressants** | | | | | | | | | | | | | | | | | | | | |
| citalopram (N06AB04) | | 275 | | 59 | | 7450 | | 59 | | 0.823 | | 0.992 | | 82.3% | | 0.8% | | 0.81 (0.78-0.84) | | |
| clomipramine (N06AA04) | | 20 | | 5 | | 7812 | | 6 | | 0.800 | | 0.999 | | 76.9% | | 0.1% | | 0.78 (0.65-0.91) | | |
| **duloxetine (N06AX21) *** | | **14** | | **9** | | **5420** | | **16** | | **0.609** | | **0.997** | | **46.7%** | | **0.2%** | | **0.53 (0.34-0.71)** | | |
| fluoxetine (N06AB03) | | 17 | | 9 | | 7807 | | 10 | | 0.654 | | 0.999 | | 63.0% | | 0.1% | | 0.64 (0.47-0.80) | | |
| mirtazapine (N06AX11) | | 139 | | 17 | | 7627 | | 60 | | 0.891 | | 0.992 | | 69.8% | | 0.2% | | 0.77 (0.72-0.82) | | |
| **paroxetine (N06AB05) *** | | **22** | | **5** | | **5416** | | **16** | | **0.846** | | **0.997** | | **48.9%** | | **0.1%** | | **0.62 (0.47-0.76)** | | |
| sertraline (N06AB06) | | 91 | | 20 | | 7710 | | 22 | | 0.820 | | 0.997 | | 80.5% | | 0.3% | | 0.81 (0.75-0.86) | | |
| venlafaxine (N06AX16) | | 79 | | 17 | | 7722 | | 25 | | 0.823 | | 0.997 | | 76.0% | | 0.2% | | 0.78 (0.72-0.85) | | |
| ***Total antidepressants*** | | ***657*** | | ***140*** | | ***59339*** | | ***221*** | | ***0.824*** | | ***0.996*** | | ***74.8%*** | | ***0.2%*** | | ***0.78 (0.76-0.80)*** | | |
| **Antiepileptics (mood stabilizers)** | | | | | | | | | | | | | | | | | | | | |
| carbamazepine (N03AF01) | | 94 | | 44 | | 7682 | | 23 | | 0.681 | | 0.997 | | 80.3% | | 0.6% | | 0.73 (0.66-0.79) | | |
| gabapentin (N03AX12) | | 14 | | 9 | | 7777 | | 43 | | 0.609 | | 0.995 | | 24.6% | | 0.1% | | 0.34 (0.17-0.52) | | |
| lamotrigine (N03AX09) | | 27 | | 3 | | 7804 | | 9 | | 0.900 | | 0.999 | | 75.0% | | 0.0% | | 0.81 (0.71-0.92) | | |
| **pregabalin (N03AX16) *** | | **44** | | **20** | | **5342** | | **53** | | **0.738** | | **0.991** | | **31.6%** | | **0.1%** | | **0.44 (0.32-0.56)** | | |
| topiramate (N03AX11) | | 9 | | 0 | | 7832 | | 2 | | 1.000 | | 1.000 | | 81.8% | | 0.0% | | 0.90 (0.76-1.03) | | |
| ***Total antiepileptics*** | | ***175*** | | ***67*** | | ***38829*** | | ***144*** | | ***0.723*** | | ***0.996*** | | ***54.9%*** | | ***0.2%*** | | ***0.62 (0.57-0.67)*** | | |
| **Antipsychotics** | | | | | | | | | | | | | | | | | | | | |
| clozapine (N05AH02) | | 19 | | 1 | | 7823 | | 0 | | 0.950 | | 1.000 | | 100% | | 0.0% | | 0.97 (0.92-1.02) | | |
| **haloperidol (N05AD01) *** | | **18** | | **7** | | **5422** | | **12** | | **0.720** | | **0.998** | | **60.0%** | | **0.1%** | | **0.65 (0.50-0.81)** | | |
| olanzapine (N05AH03) | | 59 | | 13 | | 7736 | | 35 | | 0.819 | | 0.995 | | 62.8% | | 0.2% | | 0.70 (0.62-0.79) | | |
| quetiapine (N05AH04) | | 19 | | 3 | | 7805 | | 16 | | 0.864 | | 0.998 | | 54.3% | | 0.0% | | 0.66 (0.51-0.81) | | |
| ***Total antipsychotics*** | | ***115*** | | ***24*** | | ***28786*** | | ***63*** | | ***0.827*** | | ***0.998*** | | ***64.6%*** | | ***0.1%*** | | **0.72 (0.67-0.78)** | | |
| **Cardiovascular drugs** | | | | | | | | | | | | | | | | | | | | |
| **atenolol (C07AB03) *** | | **110** | | **23** | | **5187** | | **139** | | **0.827** | | **0.974** | | **44.2%** | | **0.4%** | | **0.49 (0.43-0.56)** | | |
| **bisoprolol (C07AB07) *** | | **76** | | **16** | | **5179** | | **188** | | **0.826** | | **0.965** | | **28.8%** | | **0.3%** | | **0.41 (0.33-0.49)** | | |
| **metoprolol (C07AB02) *** | | **241** | | **53** | | **4677** | | **502** | | **0.820** | | **0.903** | | **32.4%** | | **1.1%** | | **0.42 (0.37-0.47)** | | |
| **propranolol (C07AA05) *** | | **26** | | **7** | | **5363** | | **63** | | **0.788** | | **0.988** | | **29.2%** | | **0.1%** | | **0.42 (0.29-0.56)** | | |
| verapamil (C08DA01) | | 20 | | 8 | | 7801 | | 15 | | 0.714 | | 0.998 | | 57.1% | | 0.1% | | 0.63 (0.48-0.78) | | |
| ***Total cardiovascular drugs*** | | ***473*** | | ***107*** | | ***28207*** | | ***957*** | | ***0.816*** | | ***0.967*** | | **33.1%** | | **0.4%** | | **0.46 (0.42-0.49)** | | |
| **Sedatives (as needed)** | | | | | | | | | | | | | | | | | | | | |
| alimemazine (R06AD01) | | 81 | | 62 | | 7661 | | 39 | | 0.566 | | 0.995 | | 67.5% | | 0.8% | | 0.60 (0.53-0.68) | | |
| hydroxizine (N05BB01) | | 63 | | 21 | | 7661 | | 98 | | 0.750 | | 0.987 | | 39.1% | | 0.3% | | 0.50 (0.41-0.59) | | |
| promethazine (R06AD02) | | 39 | | 15 | | 7764 | | 30 | | 0.722 | | 0.996 | | 56.5% | | 0.2% | | 0.63 (0.52-0.73) | | |
| propiomazine (N05CM06) | | 234 | | 67 | | 7337 | | 205 | | 0.777 | | 0.973 | | 53.3% | | 0.9% | | 0.61 (0.57-0.66) | | |
| zolpidem (N05CF02) | | 95 | | 27 | | 7524 | | 197 | | 0.779 | | 0.974 | | 32.5% | | 0.4% | | 0.44 (0.37-0.51) | | |
| *Total sedatives* | | *512* | | *192* | | *37 947* | | *569* | | *0.727* | | *0.985* | | 47.4% | | 0.5% | | 0.56 (0.53-0.59) | | |
| **All substances** | | | | | | | | | | | | | | | | | | | | |
| ***Total all substances*** | | **1932** | | **530** | | **193108** | | **1954** | | ***0.785*** | | ***0.990*** | | **49.7%** | | **0.3%** | | ***0.60 (0.59-0.62)*** | | |

† ATC: Anatomical Therapeutic Chemical
*** Method T1, Substances were included only for the period 2011-09-01--2013-12-31 due to the absence of routine testing at other times.**
